# Mechanotransduction In Vivo by Repeated Talin Stretch-Relaxation Events Depends upon Vinculin

**DOI:** 10.1371/journal.pbio.1001223

**Published:** 2011-12-20

**Authors:** Felix Margadant, Li Li Chew, Xian Hu, Hanry Yu, Neil Bate, Xian Zhang, Michael Sheetz

**Affiliations:** 1 Research Centre of Excellence in Mechanobiology, National University of Singapore, Singapore; 2 Department of Biochemistry, University of Leicester, Leicester, United Kingdom; 3 Department of Biological Sciences, Columbia University, New York, New York, United States of America; Harvard Medical School, United States of America

## Abstract

Mechanotransduction is a critical function for cells, in terms of cell viability, shaping of tissues, and cellular behavior. In vitro, cellular level forces can stretch adhesion proteins that link extracellular matrix to the actin cytoskeleton exposing hidden binding sites. However, there is no evidence that in vivo forces produce significant in vivo stretching to cause domain unfolding. We now report that the adhesion protein, talin, is repeatedly stretched by 100–350 nm in vivo by myosin contraction of actin filaments. Using a functional EGFP-N-Talin1-C-mCherry to measure the length of single talin molecules, we observed that the C-terminal mCherry was normally displaced in the direction of actin flow by 90 to >250 nm from N-EGFP but only by 50–60 nm (talin's length in vitro) after myosin inhibition. Individual talin molecules transiently stretched and relaxed. Peripheral, multimolecular adhesions had green outside and red proximal edges. They also exhibited transient, myosin-dependent stretching of 50–350 nm for 6–16 s; however, expression of the talin-binding head of vinculin increased stretching to about 400 nm and suppressed dynamics. We suggest that rearward moving actin filaments bind, stretch, and release talin in multiple, stochastic stick-slip cycles and that multiple vinculin binding and release cycles integrate pulling on matrices into biochemical signals.

## Abstract 2

The focal adhesion protein talin undergoes cycles of stretching and relaxation in living cells, suggesting a role in the transduction of mechanical into biochemical signals.

## Author Summary

How are mechanical forces that act on the surface of a cell transformed into biochemical signals within the cell? Studies of isolated proteins suggest that some of them can stretch, but whether this also happens in living cells remains unclear. In this study, we have been able to measure the stretching of single molecules of a cellular adhesion protein called talin in vivo by tagging each end of the protein with a different fluorescent marker and observing changes in the distance between the two markers with a new microscopic method. Talin is a large cellular protein that concentrates at sites where the cell attaches to the substratum and links integrins in the cell membrane to the actin filament network in the cell. In our study, a green tag at the integrin-binding site was close to the cell surface, whereas a red tag at the actin-binding site was displaced inward by actin flow. We observed repeated protein stretching to 5–8 times the native protein length and relaxation linked to the transduction process in living cells in culture. Individual molecules stretched for 6–16 seconds over ranges of 50–350 nm. Cell adhesion sites, where hundreds of talin molecules were displaced in concert, had similar dynamics. These cycles of stretching and relaxation required the contractile protein myosin. The head domain of vinculin—an adhesion site protein that binds strongly to the stretched talin—kept the adhesions stretched and blocked large oscillations in length. These observations indicate that there is repeated stretching of talin, and that adhesion proteins play a role in the transduction of mechanical signals into biochemical signals through binding and release of vinculin and possibly other focal adhesion proteins.

## Introduction

The transduction of cellular forces and substrate rigidity into a biochemical signal is a critical step in the control of cell viability and differentiation as well as the regulation of tissue and cell morphology [1],[2]. Several recent studies have shown that stretching of proteins in vitro can produce a biochemical change either by uncovering tryosine phosphorylation sites in p130Cas [3] or vinculin binding sites in talin [1]. Stretching of detergent extracted cytoskeletons increases adhesion protein binding [4], activates Rap1 GTP formation through a tyrosine phosphorylation pathway [5], and increases tyrosine phosphorylation levels. While indirect evidence of in vivo stretching in heat-shock responses [6] and the exposure of buried cysteines [7] has been shown and in vivo force measurements have been made on single vinculins [8], there has been no quantitative measure of the strain of proteins in vivo. The extracellular matrix is under significant stress, and the strain of Fibronectin [9] has been established in a fluorescence resonance energy transfer (FRET) assay but there is not a quantitative measure of protein stretching in cells. This raises the question of whether protein stretching (domain unfolding) plays a physiological role in the normal sensing of the mechanical microenvironment. The extent of stretch and the length of time in the stretched state provide important constraints on any model of mechanotransduction.

Measurement of the N- to C-terminal length of proteins in cells is difficult since there is normally overlap of many molecules in adhesion structures and most of the proteins have molecular lengths that are much greater than can be measured by FRET techniques (FRET is effective for 5–7 nm). Super-resolution techniques like PAL-M and STED do not enable the resolution of the N- and C- terminal positions of individual molecules in dense regions, and STED does not offer the possibility of single molecule and dual tag observation. We postulated that the presence of different fluorophores on the N- and C-termini of proteins could be used to measure the protein length in situ at both the single molecule (low expression) and multi-molecular levels (higher expression in focal adhesions) with new image processing techniques. In the case of talin, the unstretched molecule has an overall length of 51 nm [10] in vitro, and it contains up to 11 hidden, putative vinculin binding sequences [11]. The 2,000 amino acid rod of talin could theoretically stretch to a length of over 800 nm, but it is unknown whether significant stretch occurs in vivo.

## Results

**Figure 1:**
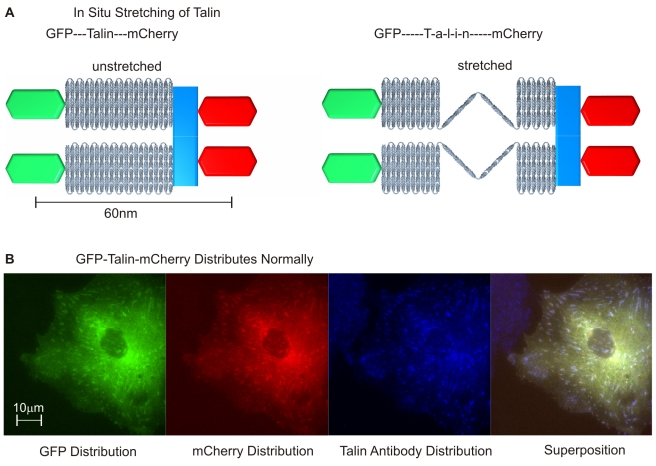
EGFP-Talin1-mCherry as a tool to measure talin 1 length in vivo. (A) Schematic of stretching of labeled Talin molecules: unfolding from a 51 nm length in the relaxed state. This can be measured in focal adhesions under stretch where the separation of the termini is apparent in two channel TIRF microscopy. Using the same edge detector criterion for both the GFP and mCherry signal reveals the dislocation of the ensemble. (B) Verification that EGFP-Talin1-mCherry distributes as expected for talin. Distribution of EGFP, mCherry, and antibody (alexa 647) staining shows colocalization of the modified and the endogenous talin (green for EGFP, red for mCherry, and blue for alexa 647 in the superposition panel) (this is under conditions of high expression but the same colocalization is observed at low expression).

A talin1 construct fused to EGFP at the N-terminus and mCherry at the C-terminus was developed and expressed in CV1 cells (diagram in Figure 1A). The distribution of the dually tagged protein was indistinguishable from the distribution of the endogenous talin 1 as determined by antibody staining of both low (not shown) and high expressing cells (Figure 1B). Another criterion for the proper behavior of the tagged molecule was that the photobleaching recovery rate of the dually tagged talin was similar to the rate previously measured, i.e. the half-time of photobleaching recovery was greater than 100 s in both cases [12]. Activation of β1 integrin was found to be unaffected by the transfection of dually tagged talin 1 in talin1 knockout cells as shown in Figure S1. Finally, dually tagged talin was able to restore normal spreading, polarization, and vinculin adhesion formation in talin-depleted cells [13]. Thus, the presence of the two fluorophores did not alter talin binding, function, or its dynamics in adhesion sites and ruffle movement in any detectable way (Figure S2).

**Figure 2:**
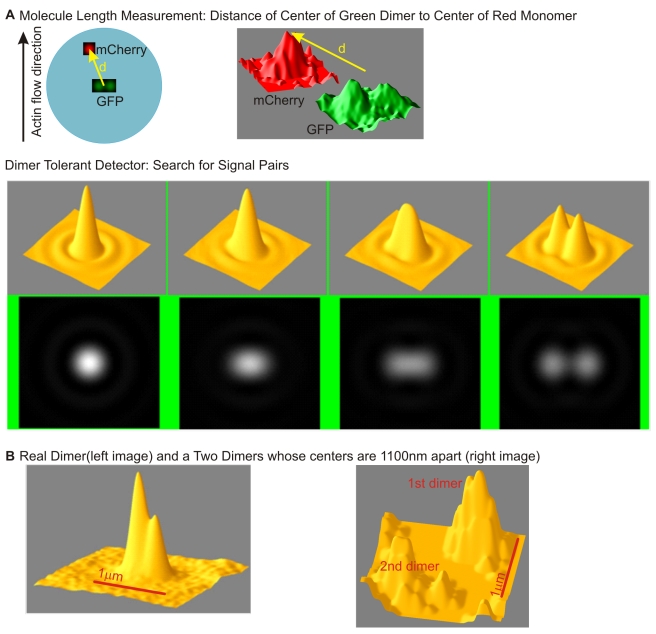
Single molecule signal processing. (A) Dual centroid tracking: If the Talin complexes are isolated by 500 nm from other fluorophores, the location of the EGFP and the mCherry markers can be determined with a single frame accuracy of ±20 nm with a variance of less than 14 nm as statistical analyses showed. For EGFP the center of the dimer is calculated. The right slide shows the clarity of the fluorescence signal with two separated EGFPs and one mCherry spot. Centroids of the deconvolution are tracked, and orientation and displacements of the EGFP and mCherry pairs are measured. The orientation vector is defined as the line from the average EGFP position and the mCherry position. Dimer tolerance is built into the detection system. The dual EGFP signals cannot be safely identified by a simple centroid or PSF maximum likelihood technique, and the exact shape of the signal was matched to two closely spaced PSFs. In order to keep noise and random constellation responses low, the exact allowable shape of the dimer PSFs was defined, and the detection algorithm tested the sample against these PSFs. Simply making the detection PSF broader resulted in an unacceptable level of false positives in our pre-processing. By basing the detection on dimers rather than on isolated fluorochromes, dimers can be safely identified in close proximity to other dimers. Unless the PSF of a dimer ruins the shape detection of its neighbor, the latter can be identified reliably. The centroids of these two dimers are separated by about one micron. (B) With increasing expression density, the probability of falsely accepting the C-terminal of one molecule as linked to the N-terminal of another one increases (density artifact). In this trivial case, the collision would, however, be detected by the filter. But if the EGFP tags on one molecule were not fluorescent, the error would not be flagged by the software.

**Figure 3:**
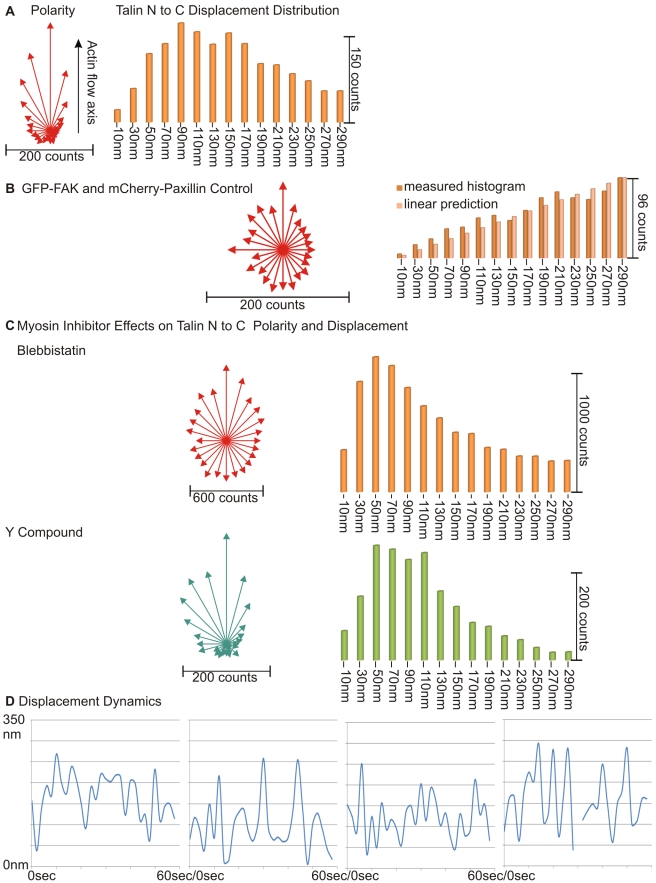
Single molecule analysis shows orientation and stretching of talin. (A) Polarity and displacement of Talin N- and C-termini: All clear single molecule pairs in a field of view over time are analyzed. Each molecule contributes length and angle information for a single count in the histogram in each time frame. Fast changing molecules hence contribute different lengths and orientation information at different times. We used an average of 10 time steps for these observations. The polarity histogram segments the full circle into 24 sectors of 15 degrees each, and the number of vectors that fall in a given direction is represented in the length of the vector. This coarse version of a scatter plot is chosen because it is robust against small angular variations due to the error when estimating the direction. The direction is measured relative to the previously established orientation of the actin flow. The displacement distribution counts how many vectors fall in each length range of 20 nm width. The 10 nm “bucket” ranges from 0 to 20 nm, the 30 nm ranges from 20 to 40 nm. etc. (B) Polarity and displacement of EGFP-FAK and mCherry-Paxillin control: In a cell where there was roughly equal expression of EGFP and mCherry, there was weak orientation of the EGFP-mCherry vector, and the histogram plot of the separation distance rose linear with radius as expected for unlinked molecules. The likelihood to find a partner at a distance is proportional to the area at that distance and the observation corroborates this. These conclusions only hold for the low expression case. In Figures S3 and S4, we add simulator data to show how the algorithm assesses that the measurement falls within a valid range. (C) Effect of myosin inhibition on polarity and displacement: Myosin inhibition with blebbistatin resulted in a relatively uniform molecular orientation histogram, although adhesions in blebbistatin had a clear orientation over the observation period. The displacement histogram has a clear, non-zero peak at about 50–60 nm. After inhibition of Rho kinase with Y-27632, the N- to C-terminal vectors are oriented, and the displacement histogram has peaks at 50–60 and 110 nm. (B) Without inhibitors, the orientation becomes much clearer and the stretching longer. (D) Dynamic stretch cycle of a single talin observation: In specimens with low background, excitation energy can be kept low and observations over several frames are possible, allowing for insight into the repetitive stretching cycle of Talin. Most of the traces—i.e., a single molecule that was observable over several frames—live between 4 and 15 frames but we found red-green pairs being stable for more than 30 frames (with a current score of 67 frames). At 2 s observation intervals, this allows for the plots shown here. Not all molecules can be tracked reliably in all frames, which leads to dropouts which are also shown here. In nearly all observed cases, the EGFP tags here are at rest within the accuracy of the method, whereas the mCherry signal exhibits motion. In the specimen we investigated, between 2% and 30% of all pairs evolve over time and can hence be used for stretching observations. We do not know what causes high and low yield in terms of observable stretch. Specimen more at rest were easier to observe and kept focus tough. The interesting feature here is the highly regular oscillating moving pattern that suggests a rapid relaxation as well as stretching of the molecule.

As a further test that the chimeric protein was behaving normally, we measured the fluorescence characteristics of individual proteins in low expressing cells. Our criterion for a single talin dimer was that two EGFP molecules were separated by more than 500 nm from other EGFPs (10 times the in vitro length of talin), and that there were two bleaching steps in the green channel and at least one in the red channel (previous studies have noted that only about 50% of mCherry molecules will be fluorescent upon expression in mammalian cells [14]). In cells with low expression and well-separated fluorophores, we typically observed dimeric EGFP spots (Figure 2A). These were dimeric based upon two criteria: (1) they normally bleached in two steps and (2) they were not normally detected by an algorithm to fit a single point spread function (only 15% of the isolated EGFPs were detected with a circular PSF and in some cases the two fluorophores were well separated) (also Figure 2A). In the case of the mCherry fluorescence, the fluorophores were normally detected by a circular PSF (80% of fluorophores), and there were fewer double bleaching events so that we typically linked individual mCherry fluorophores with the dual green fluorophores. When the distance between the green dye pairs and red dye was measured for linked fluorophores, the green-red vector was typically oriented in the direction of the actin movement (Figure 3A) and the displacement histogram plot showed a global maximum at 90 with a secondary peak at 150 and shoulders at 50 and 210 nm. As a control, we co-transfected cells with two unlinked focal adhesion proteins, EGFP-FAK (focal adhesion kinase) and mCherry-paxillin, and performed the same analysis (Figure 3B). However, the EGFP–mCherry axis did not show strong orientation and the number of observed events increased linearly with displacement as expected for a random distribution (the sampled area increases linearly with radius). When we went back and fit the observed normal histogram for EGFP-talin-mCherry using the known density of fluorophores in the region and the fraction of unfolded mCherry molecules, we found that the random interference of the adjacent molecules contributed significantly to the mis-aligned vectors in the normal talin1 plot and to the histograms at large displacements (notice that at low expression, the expected number of random events should scale proportionally with area and there is a linear increase in the area sampled with increasing radius). Even after correction, these measurements show that talin molecules are stretched in the direction of actin movement in vivo.

If the stretching of the talin molecules was due to myosin contraction of the actin filaments, then inhibition of cellular contractile activity might inhibit orientation and displacement [15],[16]. After blebbistatin addition to inhibit myosin contraction, there were two major changes at the single molecule level: (1) the orientation of the green-red dipoles was random and (2) the average displacement was much less, giving a peak at 50–60 nm (Figure 3C, top). If the cells were treated with Y-27632 to inhibit the Rho Kinase, traction forces were dramatically decreased but peripheral contractions persisted (Figure 3C below) [17],[18]. Single talin dimers were still oriented, but the level of displacement decreased giving a major peak at 50–60 nm and a secondary peak at 110 nm. The major peak in both inhibitors was consistent with the length of the in vitro talin molecule, i.e. 51 nm (assuming that 5–10 nm should be subtracted for the linkers and the fluorescent proteins) [10]. The longer forms potentially indicated that some of the bound molecules were stretched but correction for random events removed most of the cases above 200 nm. This was consistent with the fact that about 20% of normal force was generated even in the presence of blebbistatin [15].

We wondered if the stretch was static or changed with time. In several cases, we were able to follow isolated EGFP-mCherry pairs over 30 to 60 s at 2 s intervals. Clear changes were observed in the length of single molecules over a time frame of 10–20 s, but there was significant randomness in the stretching events (Figure 3D). We estimated the variance of the position measurements for a single frame to be ±14 nm from a statistical analysis of multiple position measurements of the EGFP fluorophores that did not show any directed movement. Thus, we found that there was significant stretching of single molecules in a transient manner (see also Figure S3, which provides the corresponding micrographs and pair locations).

**Figure 4:**
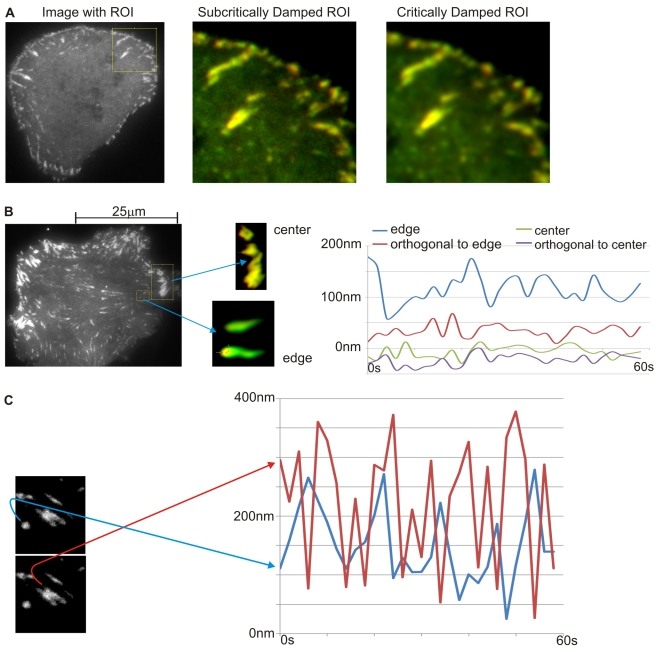
N- and C-terminal displacements in adhesions. As the individual molecules are small compared to extension of an adhesion and the stretch small compared to the optical resolution of the microscope, it is difficult to observe the direct stretch of dually tagged molecules. The red-green shift induced by oriented stretch can only be resolved if the adhesion is optically isolated so that the centroid of the edge in each channel can be determined. The multimolecule algorithm covers the case where the expression density is high and the adhesions are clearly visible on a dark background. The observed shift between the green and the red channel is the ensemble (average) stretch of the molecules at the periphery of the adhesion. The ensemble stretch cannot be resolved as a direction; hence, we always measure it along the long axis of the adhesion. The uncertainty of direction and the averaging of the ensemble are the shortcomings of the method, the ease of the experiment its major advantage. (A) Fluorescence distribution and image processing: After compensating for chromatic aberration, peripheral focal adhesions were typically green at the outer edge and red at the inner edge in the Region of Interest (ROI). If the ROI was processed with an incorrect too large point spread function (PSF) as in the center panel, the boundaries of the adhesion complexes were unstable from frame to frame and that hindered displacement measurements. In the right image, a well adjusted PSF was used in the processing, enabling measurement of the displacement of the different fluorophores (details are in the Materials and Methods section). We mention this because acquiring the correct PSF in such an arrangement is a challenge and did require planting of sub-diffraction sized beads in the specimen as calculated estimates proved not stable for this purpose. (B) Peripheral adhesions show greater displacements over time: An active adhesion at the edge (showing a green edge at the right outer region and red edge at the left boundary, itsinner region) and a central adhesion (showing no such trend) are highlighted in the ROI. Plots of the green edge to red edge displacements versus time are presented with+being in the direction of the centripetal actin flow for the edge adhesion and toward the nucleus in the central adhesion (for the orthogonal plots, the +axis is +90°). The edge adhesion shows the strongest displacement and the largest oscillations, the central one has a small average displacement, and the orthogonal displacements are smaller than the axial displacements. (C) Adjacent adhesions move independently: These two adjacent adhesions have large displacements that are uncorrelated. The adhesions are located some 6 micrometer from the leading edge. Myosin inhibition blocks movement: (A) After blebbistatin inhibition of myosin, talin displacements are at the level of noise. After Y-27632 inhibition of Rho kinase, talin displacements are diminished to 40–60 nm, consistent with the single molecule measurements. The dynamic blebbistatin measurements did not prove to be significant in the statistical test. Movements are too shallow and the oscillations too regular to raise the likelihood above the stochastic model, with the exception of the datasets in which the suppression is not perfect. This means that the residual small erratic movements do not necessarily constitute physical reality of the molecular orientation. The negative result in itself, however, is significant: the observed suppressed molecules are at rest within the accuracy of the method. (B) Vinculin head promotes talin stretching and suppresses dynamics.

To be able to better measure the dynamics of the N- to C-terminus displacement of talin in situ, images from cells with higher expression levels were analyzed giving an average displacement. Adhesions with greater fluorescence intensity than 200 fluorophores were chosen. We found that after correcting for chromatic aberration, the mCherry signal was predominantly displaced toward the nucleus from the EGFP signal in peripheral adhesions (Figure 4A & B, multimolecule analysis), whereas in central adhesions, the displacement was usually significantly less. As a control, we analyzed the displacement of mCherry and EGFP in cells expressing both EGFP-paxillin and vinculin-mCherry that should localize randomly in adhesions. In that case there was no preferential direction and no changes were observed over time (Figure 3B, single molecule analysis). To objectively determine the displacement over time, we analyzed each region of interest (ROI) with an iterative constraint deconvolution algorithm that created a super-resolved map of the image based upon the PSF of the microscope (see Materials and Methods) and a direct, reduced derivative of the Richardson-Lucy Deconvolution [19],[20]. The intensity of the fluorescence in the adhesion did not require the fluorophores contributing to each point image to be close to the average intensity, but the signal density needed to be high enough such that the point images of the fluorophores overlapped. Within the map, the edge was then found via a conventional Canny edge detector (Figure 4A) [21]. When the displacement between the green and red leading edges was measured (the edges toward the periphery), the displacement changed over time, rapidly increasing to as much as 350 nm of separation and dropping to near zero (Figure 4B). Our estimated error of the measurement was ±20 nm and measurements of displacements orthogonal to the axis of actin movement often had fluctuations of ±15 nm over time. In cases where the direction of actin flow was measured, the mCherry fluorescence and presumably the C-terminus of talin were always displaced with the actin flow toward the nucleus. This was true for adhesions on the opposite sides of the same cell or ones oriented at right angles to each other. The average time for the large excursions of the C-termini was of the order of 6–16 s and was much more rapid than the photobleaching recovery rate for talin, indicating that talin was stretched and released multiple times. When two adhesions in the same field were analyzed, they often were uncorrelated in their movements (Figure 4C). The displacements in the round adhesion were relatively slow and had an average lifetime of 13 s, whereas the displacements in the adjacent longer adhesion were more rapid (also Figure 4C). Stress fibers in these cells were normally attached to longer adhesions. The stochastic nature of the displacements was consistent with a transient attachment of the C-terminal to rearward moving actin, displacement, and release, as in a stick-slip coupling.

**Figure 5:**
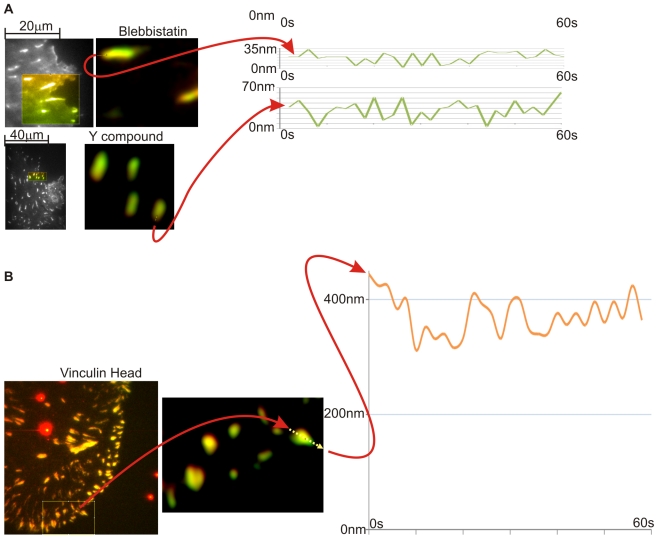
Displacements after suppression. (A) Blebbastatin and Y-compound: Time evolution of the stretching after adding Blebbastatin or Y-27632, respectively. The total displacement and the amplitude of the stretching are decreased very noticeably. Even though for the Y-27632, it is still clearly larger than the error of observation. (B) Vinculin head added: The vinculin head construct stretched out the Talin molecule and left it stretched. There are still observable oscillations in stretching, but the amplitude of change is dramatically suppressed.

When inhibitors of contraction were added, the displacements in the adhesions decreased dramatically (Figure 5A). Although adhesions persisted in the presence of blebbistatin, the displacements were barely above background levels (displacements of 0–30 nm). In the presence of the Rho kinase inhibitor, the fluctuations decreased dramatically from the normal case (displacements of 0–50 nm, lower plot, Figure 5A). This was consistent with the decreased contractile activity and decreased actin flow rate that should have resulted in smaller displacements before the release and slip.

From the steered molecular dynamics analyses of the talin rod, the stretching of talin by 100 to 200 nm could reveal 5–7 vinculin binding sequences [22],[23]. This is in line with the presumed function of talin stretch to increase the number of bound vinculin molecules. Upon relaxation of talin, the number of bound vinculin molecules should decrease. Thus, the timetable of stretching and relaxation may be related to the exchange rate of vinculin in adhesions. Other studies have shown that vinculin has a much shorter lifetime in the adhesions than talin (t_½_ of 16 versus >100 s) [24],[25] and the vinculin lifetime is close to the time of the stretch-relaxation cycle that we observe. It is possible, therefore, that the expression of the vinculin head that binds tightly to talin would cause a decrease in adhesion dynamics and promote talin stretching. In cells transiently expressing a vinculin head construct, there was a dramatic increase in the displacement of the C-terminal mCherry from the N-EGFP to levels of 400 to 600 nm and a suppression of the dynamics of the length change (Figure 5B). In normal cells, the level of endogenous vinculin staining in adhesions correlated well with the level of talin, N- and C-terminal displacement (Figure S4). In the case of activated FAK staining [26], there was a weak correlation of staining intensity and the level of displacement (Figure S5). Because the strong binding of activated vinculin head caused increased talin stretching with less dynamics and increased endogenous vinculin staining correlated with increased stretching of EGFP-talin-mCherry, we suggest that talin stretching in vivo results in vinculing binding.

## Discussion

**Figure 6:**
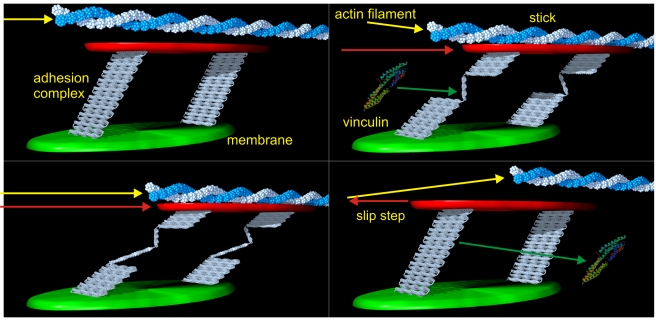
Stick-slip mechanism. Cartoon of the suggested mechanism. The rearward moving actin filament (yellow arrow) binds to talin's C-terminus and exerts a mechanical pull on the adhesion. Talin will stretch via unfolding in steps that enable vinculin binding until the slip bond releases. At this stage, the filament slips, the talin refolds with the release of vinculin, and the process starts anew. The vinculin image is property of the Protein Database.

The EGFP-talin-mCherry protein appears to be a good reporter of talin1 function and the stretch of talin in vivo. The measured lengths of the talin molecules of 80 to 350 nm are consistent with the structure of the talin rod (roughly 2,000 aa in the rod that could be stretched to a length of 800 nm) and the dependence of the stretch upon contraction is consistent with its dependence upon actin flow driven by actomyosin pulling. In the single molecule case, the displacements of the two EGFPs are well within the expected range for the splayed ends of a parallel dimer that is linked at the other end [11]. In the multi-molecular analyses, it is surprising that the N- and C-terminal ends of talin in the adhesions are displaced from each other since it implies that there is a lateral aggregation of the N-termini distinct from the C-termini. Recent analyses of talin using iPALM showed that the talin N-termini were at the membrane surface and C-termini were 30 nm above the membrane [27], but they were unable to measure simultaneously the N- and C-termini to estimate the average length of the molecule. Based upon those results and the findings here, it appears that the two ends of the talin molecules are located in different complexes that are part of focal adhesions (as drawn in Figure 6). The fact that the displacement oscillates over time in a myosin-dependent manner rules out many possible artifacts of the measurement system, particularly since myosin-inhibited samples fluctuate 5- to 10-fold less over time. Thus, we suggest that the centripetally moving actin (moving typically at 40–60 nm/s) transiently attaches to the C-terminal talin complex that may include other adhesion proteins, stretches talin, and then releases it.

The linkage between the actin cytoskeleton and the adhesion complexes generating traction force has been treated as a clutch [28] that generates traction force before slipping in neurons [29]. However, fibroblasts behave differently from growth cones in that there is slower actin flow and greater force on rigid surfaces [30],[31]. The time scale of force generation at the level of even sub-micrometer adhesions (Ghassemi, Sheetz, and Hone, personal communication) is considerably longer than the oscillations in talin length either at the single molecule or adhesion level. Thus, we suggest that talin stretching occurs stochastically through a transient slip bond between inward moving actin filaments (Figure 6) and talin C-terminal complexes that contributes only secondarily to the overall traction force. Logically, a bond between talin and actin cannot be maintained for very long because the velocity of the actin movement rearward is 40–60 nm/s and the lifetime of talin in the adhesions is over 100 s, giving potentially 4,000–6,000 nm of stretch. In the case of the adhesion complexes, there appears to be a concerted release of the talin C-termini from the actin (there are several hundred talins in each adhesion and the displacement is rapidly lost). Two possible explanations are (1) enzymatic modification or (2) a concerted release by a fracture phenomenon such that the breaking of the bond under the highest force passes that force to the next bond causing it to break rapidly resulting in all bonds breaking in rapid succession. In either case, we consistently find a concerted displacement of the talin C-termini in adhesion complexes in the direction of actin flow that is dependent upon myosin contraction. The dramatic oscillations in the length of the talin molecules are inconsistent with the stable nature of forces on pillars that only oscillate on the time scale of minutes and not seconds as does talin stretching. Thus, we postulate that the major role of actin stretching of talin is in signaling and not force generation.

The oscillations in talin length appear to affect the binding of vinculin since increased vinculin binding through expression of the active head appears to increase talin length as well as decrease oscillations in length. Recent studies in mice expressing a vinculin mutant that behaves like the active vinculin head find early lethality [32], which indicates that vinculin dynamics are important for cell function in vivo. These studies are also consistent with developing views of the adhesion sites as signaling complexes with peripheral and core components [33]. Cycles of binding and release could be linked also to enzymatic modifications of the bound proteins such as the phosphorylation of FAK that would enable the cells to accumulate signals from rigid matrices that would support growth and differentiation processes. Much more work is needed to understand the role that the molecular stretching of talin in vivo plays in the functions of adhesion structures and cellular behavior. However, this method for measuring the displacement has demonstrated that stretching-relaxation cycles occur in vivo as a result of myosin contraction by a stick-slip mechanism.

## Materials and Methods

### Cell Culture and Reagents

CV1 cells were used for these studies and cells were transfected with lipofectamine 200,024 hours prior to resuspension and plating onto fibronectin coated coverslips. Cells were typically imaged after overnight plating. Inhibitors of myosin were added at the time of plating. 10 uM of blebbistatin and 10 uM of Y-27632 were used in the inhibition assay, and care was taken to not expose blebbistatin solutions to light except at the time of image acquisition. Blebbistatin, Y-27632, and fibronectin were purchased from Sigma. Phospho-FAK antibodies were obtained from Invitrogen. Talin and vinculin antibodies were purchased from Sigma.

### Immunofluorescence

CV1 cells were fixed in 3% paraformaldehyde in phosphate buffered saline (PBS) for 30 min. Fixed cells were washed twice with PBS, twice with PBS containing 50 mM NH4Cl, and twice again with PBS, followed by permeabilization with 0.1% Triton-X-100 (Sigma-Aldrich) (room temperature, 15 min). Permeabilized cells were incubated at room temperature for 1 h with 30 µl of anti-Talin, anti-Vinculin (Sigma), or anti-phospho-FAK (Invitrogen) at 1∶200 dilution (in 2% fetal bovine serum, 2% bovine serum albumin, and 5% goat serum in PBS). Samples were washed three times in 0.1% Triton-X-100-containing PBS followed by two times in PBS before incubation with AlexaFluor 647 goat anti-mouse or AlexaFluor 647 goat anti-rabbit (Molecular Probe, Invitrogen) at 1∶200 dilution (in 2% fetal bovine serum, 2% bovine serum albumin, and 5% goat serum in PBS) for 1 h at room temperature. After the final wash (three times in 0.1% Triton-X-100 containing PBS and two times in PBS), coverslips were mounted with FluorSave (Calbiochem, San Diego, CA) and examined under a DeltaVision microscope (all images were captured with a 100× oil objective lens).

### Microscopy

In the stretching experiments, frame-of-reference fiducials, Zeiss 100 nm yellow beads, and 250 nm Invitrogen Tetraspeck beads were used. A stable frame of reference was deduced from fluorescent beads, which settled, stuck to the Fibronectin-coated coverslip, and did not exhibit thermal movement.

Tracking relied on dual channel time-alternating TIRF images from an Olympus 1.49 100× oil immersion system at a 1.5× relay magnification using excitation wavelengths at 488 nm with 27 mW and 561 nm with 15 mW of power, recorded on a 1024×1024 pixel Photometrics Cascade II EMCCD camera at 10 MHz readout speed, 13 mm pixel size, at an operating temperature of −42°C with external forced airflow. The imaging system was calibrated for chromatic aberration, dark current, and read noise using Zeiss 100 nm yellow beads and 250 nm Invitrogen Tetraspeck beads.

Single molecule tracking used exposure times of minimally 5 ms at 488 nm and 20 ms at 561 nm, respectively, up to 100 ms for both. The interval between channel pairs was 2 s. Signal molecule tracking was achieved by fast correlation followed by adapted maximum likelihood (referred to as ML henceforward) single PSF fit, tuned to the dimer signal for the EGFP signal and mild defocus PSF for the mCherry channel. The ML parameter of choice was how well the photon statistics of a single fluorophore (forming the point source) added to a locally constant background of defocused light, and could explain the brightness observed. The center coordinates of the point sources are moved until a local maximum in likelihood is reached. We moved the center in three dimensions, hence allowing for a mild defocus of the center coordinate. Pair occurrences of both fluorophores within a 300 nm radius (the “catching radius”) region were recorded as single molecule candidates.

Since we used both excitation lines at the same TIRF angle, we had to use averaged and hence relatively tolerant TIRF settings. Also, since the adhesions form areas of elevated refractive index, some widefield leak could not be prevented. The detector and localization software worked well with these settings, since the point-spread functions of the single molecules were very clear and discernible with highest accuracy.

### Microscopy of Single or Few Fluorophores

The EGFP signals were usually dimers whose components were too close to be resolved by the microscope (assuming the 250 nm Abbe limit of our oil immersion system) but generally too far apart to result in the image of a single point. Assuming that the PSFs were dimeric (Figure 2A), the detector tested several dimeric PSFs to determine the best fit of each molecule (Figure 2B). Merely widening the accepting shape of the PSF enabled efficient detection of the dimer signals, but it resulted in accepting significantly more background (i.e., random noise constellations). Hence, PSF widening was abandoned for our image processing, although it was an order of magnitude more efficient in computing time.

The mCherry PSF appeared elevated above the cover slip when the microscope was focused on the GFP fluorophores at the glass surface. The detector was defocused in 10 nm steps for up to 50 nm to test for the defocus of the mCherry monomer. For acceptance, the mCherry signal needed to be within the catching radius of the EGFP signal and to be active for several frames (usually four or more). This temporal co-localization requirement acted as strong noise rejection filter, eliminating about two in three signals that passed the PSF shape criterion. With increasing pull, the mCherry molecules tended to be closer to the coverslip resulting in cleaner PSFs.

The observed signal levels of the two fluorophores differed dramatically: mCherry lived far fewer excitation cycles than EGFP (above 20K cycles versus 40K cycles before average bleaching), the mCherry signal was lost due to the defocus blur, and fewer mCherry molecules folded properly (35% folded properly rather than 65% for EGFP). This limited the observable number of frames but did not impact the detection accuracy in any measurable way. Both the distortion from the dimer and the defocus of the mCherry monomer were learned by the detector through empirical tests.

The main challenge regarding the image processing was that it proved virtually impossible to create expression levels that were low enough to be analyzed by photoactivation localization microscopy (PALM) software. The lowest meaningful molecular density was a good order of magnitude larger than the density that was acceptable to the fast filters used for the PALM correlation detection. Thus, the PSFs of many molecules partially overlapped or collided. Being able to deal with overlapping PSFs was a prerequisite to design an algorithm with an acceptable yield (example depicted in Figure 2B).

### Single Fluorophore Image Processing

To compensate for the problems with the focus and overlap, we used computationally very inefficient matched filters that were trained with our own datasets. The PSFs used for the detection algorithm were harvested from sparse test datasets whose centroid signals were the most radially symmetric we could find and with minimal blur (i.e., they needed to be in focus). 10–40 such PSFs were centered and then fused into a single PSF suitable for detection. This provided a very selective and reliable first stage filter for the EGFP tags (even for the dimeric ones) but—due to large focal range—proved unsuitable for the mCherry monomers. The search was hence executed hierarchically: (i) EGFP candidates were identified first; (ii) dimers were analyzed; (iii) pairs of dimers that collided—i.e., came too close to other valid EGFP signals within the detection range—were rejected; (iv) nearby mCherry signals were analyzed for the proper focus; and (v) mCherry collisions were rejected and (vi) tested for continuity of the EGFP-mCherry pairing for at least *N* frames (we mostly used *N* = 2 or *N* = 4 and even *N* = 2 provided an excellent rejection against noise artifacts). The matching itself was done by calculating the logarithmic likelihood that the observed signal was matched to the PSF model.

We use a computational model of the PSF to account for all the possible dimers and to implement defocus more easily. No restrictions on the signal amplitude were made, and the matching was limited to a circular region of 300 nm radius in order to still allow for dense signals. A raw micrograph of an unprocessed molecule experiencing stretching and the output of the correlation filter is presented in Figure S6.

A ML method with such restrictions is not any more accurate than a centroid detector (our localization algorithm performs considerably worse than the PAL-microscopy software in most cases), but it performs well in an environment where the signals are denser. The fraction of usable signal was raised considerably (more than a factor of 3 over the autocorrelation detector) but often still did not reach 50% of the observable speckle-like signals. We rejected specimens with a very low fraction of analyzable signals (i.e., a high number of detected collisions) due to the danger of admitting statistically non-significant data (see density “artifacts” in Figures S7 and S8).

Motion was not a serious problem for our setup since the PSF signals were reliably clean and motion artifacts would not pass the defocus tolerance. The “speed limit” for exposure times of up to 100 ms was just above the 60 nm/s of the average actin flow. Higher speeds needed a more tolerant detector (which was computationally unreasonably expensive for the follower-channel) or shorter exposure times.

Oddly enough, the single molecule pair-observation was not limited by the dramatic loss of single molecules detected when the signals become as dense as a PSF diameter. However, if we consider the number of erroneously assigned tags, at high densities, a random tag from another molecule becomes a more plausible “partner” than the true second label (Figure 3B and Figures S7 and S8). We were forced to monitor the signal density and reject samples with too great a density, since the above error cannot be corrected in our algorithm. The error can be estimated safely but not overcome if present.

### Multimolecule Tracking

At high densities, though, proper images were formed whose boundaries were defined and the edge was localized by conventional edge detection theory (Figure 4A and 4B), mainly Canny detectors, which allowed for sub-pixel localization of the boundary. This ensemble detection of dense fluorophore assemblies was further improved by subjecting it to a Richardson-Lucy deconvolved super-resolved ROI. Edge detection was limited orthogonally to the surface, which allowed for the one-dimensional integral across the entire leading edge in order to obtain a more stable readout. This method was applied to fiducial recordings. It showed a tracking accuracy and repeatability better than 25 nm for focal adhesions prior to bleaching.

### Data Processing Statistics

As both localization methods are novel, we derived a *t* test for paired samples of our measurements versus two powerful negative hypotheses: (i) the static measurements were realizations of random, normal, but spatially variant fluctuations and not actual single molecule stretching observations, and (ii) our dynamic data were the result of a systematic calibration error superimposed by a locally variable measurement noise. We assumed the worst case for all errors: that the distributions of the noise matched the observed movements optimally and that the calibration error canceled out all observations optimally. Using a powerful and universal error model was necessary to segregate our observation from all possible alternative explanations. Consequently, our confidence intervals were relatively pessimistic. For the static case, any observation shorter than 18 nm and 22 nm, respectively (the first value for the observed jitter model and the second one for an upper bound of single molecule statistical variance), was not significant compared the noise models. That is, measurements shorter than this value were possibly noise. That was about 50% worse than what the observations of our ensemble variance suggested but was a logical consequence of the test limits. The variance was at two-thirds of these values and hence only the average of the observations was truly significant—a single measurement needed to be longer than 36 nm and 45 nm (again, depending on the underlying model), respectively, to be 99% significant.

The result for the dynamic tests (Figure 3D) was more merciful solely because the distances of motion overshot the latter limit on average. But as the error model here is even more powerful, numerous observations were required to establish significance. For the dynamic measurements, only length changes in excess of 18 nm were significant over the null hypothesis: that is, the quotient of the likelihood of a single observation over the likelihood of a single random process was 1.38 for the active normal datasets and 1.32 for the active Y-27632 datasets. The dynamic data for blebbistatin was not significant at all (Figure 5A) and the dynamic measurements in the presence of blebbistatin constituted a negative result. The observed durations for molecules in our tests were 22–27 frames of 2 s each for the normal case and 22–25 frames for the Y-27632, raising the previous probability ratios to these powers. The total probability ratio per measurement of a single molecule was ∼6,000 for the normal case and ∼500 for the Y-27632 samples. The maximum separation quotient per entire experiment was 1∶10^41^ for normal versus 1∶10^50^ for Y-27632, since the latter showed an average of 18.5 long living molecules versus the 10.9 in the normal case. The observed movement of molecules over time can hence only be explained by a structured movement over time and not by a random fluctuation.

### Image Processing: Fiducial References and Chromatic Aberration

The fiducial beads were identified with a correlation filter and the local maxima above the 30% threshold of the dynamic range, in order to segregate the beads from fluorophore clusters. Beads were localized in two steps via a centroid measurement followed by a maximum likelihood fit. The latter also helped to eliminate beads that were not in contact with the coverslip or showed motion artifacts. The beads that traveled the shortest distances over the observation period were admitted to the pool of the “ensemble.” This assured that loose beads or beads attached to the cells were not taken into account for the frame of reference. The accuracy of matching was defined as the square root of the variance of a single fiducial relative to the ensemble. We obtained values of 2.8 nm for the TetraSpecks and 4 nm for the Zeiss yellow beads. Adding a maximum likelihood detector to the centroid measurements only improved the stability if the beads saturated the detector. When signals were in the linear region, no change or improvement was noticed.

Dense fields of beads without biological samples were used to determine the chromatic aberration. The yellow beads emitted broadly in both channels and hence delivered a too optimistic estimate for the lens errors. But the TetraSpecks beads nicely matched our fluorophores and hence were used for all calibrations. The same selection procedure for non-sticking beads was used. Several frames were needed to establish a dense frame of adherent beads, even though the chromatic error was a feature that changed slowly over the field of view. The field of view was tessellated into triangles by the chromatic reference. Within the tessellation, we used tri-linear interpolation to establish the chromatic shift at any given location. We used the green channels as the reference and corrected for the red coordinates. Each red measurement was complemented by the chromatic vector and hence was seen relative to the green channel. No absolute reference was required anywhere in our measurements as all distances were assessed locally.

### Image Processing: Single Fluorophore Localization

The localization of fluorophore tags—as described above—was photon limited. For both centroid and ML localization, the accuracy increased with the square root of the photon count, but the limits were about 30% narrower for ML matches. ML was also more robust against “collisions”—partially overlapping PSFs—as well as motion and defocus artifacts. The cost was a steep increase in computational effort and an actually much worse performance if wrong PSFs were used. In the best quiet datasets with Blebbistatin, the ensemble of the red fluorochromes was stable within 18 nm for the entire population and 10 nm for the strongest signals. For the brighter green fluorophores, these values were 14 nm and 8 nm, respectively.

For the active datasets, the single frame accuracy was as calculated above. However, the green frame was recorded 500 ms after the red one, which introduced a significant uncertainty in the motion prediction. While we extrapolated the frame of reference to the recording time, the terminals of stretching molecules were observed at different stages and hence the distance between the terminals was interpolated. We did this by assuming that the motion was band limited and hence used sin(t)/t for the time interpolation. At the speeds of the actin flow, the differences between the sin(t)/t interpolation and no interpolation can be as great as 8 nm with no safe way to determine which one was actually more accurate but that is a small fraction of the observed displacements.

Assuming that the reference errors, the interpolation, and the localization were independent processes, there was a ∼20 nm error for observation accuracy. If the extrapolation was free of errors, this was reduced to ∼18 nm and ∼15 nm, respectively.

This also imposed strict limits of what we regarded as significant stretch, motion, and orientation. Distances must reliably exceed the above limits, motion must either exceed or stay beyond the above limits to be identified as “real,” and both coordinates which define an orientation must consistently be separated by more than the observation limit. The orientation plots in Figure 3 require observed stretching of 60 nm or more to be quantitatively meaningful under the above limits.

## Supporting Information

Figure S1 β1 integrin activation. Talin1 knockout cells were transfected with EGFP-Talin1-mCherry, fixed, probed with an anti-activated β1 integrin antibody, and stained with a secondary AlexaFluor647 antibody. Images were collected using a Deltavision Microscope. EGFP signals were presented in green, mCherry signals in red, while the AlexaFluor647 signals were in blue.
(TIF)

Figure S2 Expression of GFP-talin1-mCherry fully rescued the cell morphology and vinculin puncta formation in talin1−/− cells expressing talin2 siRNA (talin-depleted cells [13]). Cells were fixated after 60 min of spreading and stained for vinculin. This confirms that the GFP-talin1-mCherry construct behaves like natural talin when expressed in talin knockout cells, as they were capable of forming normal adhesions that also co-localized with vinculin. 1^st^ plot, stretching of 2^nd^ pair from the top; 2^nd^ plot, stretching of a pair in the lower right; 3^rd^ plot, trace at the leading edge; 4^th^ plot, pair from the long adhesion in the center.
(TIF)

Figure S3 Talin single molecule observation over time. In Figure 3D we plot four distinct time courses of single fluorochrome pairs, each of which allowed observation over 60 s in 2 s time intervals (the arrows indicate the stretch of a pair at the snapshot time, white indicates the particular molecule we plotted, and red the average direction of all measured molecules). While GFP tags easily live in excess of hundred observations, mCherry proved to be more fragile, with the majority of the observations for an isolated pair being in the range of 15 to 20 frames. Imaging disruptions are, however, far dominant over fluorochrome blinking—if the PSF gets scattered, the frame drifts too fast, or another fluorescent signal comes too close during the observation, all this will interrupt the measurement. And even the successful observations in Figure 3D exhibit single frame drops where the localization proved not to be stable. Here we append the micrographs of the specimen from which these measurements were taken.
(TIF)

Figure S4 CV1 transfected with EGFP-Talin1-mCherry were fixed, reacted with an anti-vinculin antibody, and stained with an AlexaFluor647 secondary antibody. Images were collected using a Deltavision Microscope. Both EGFP and mCherry signals were represented in green and red, respectively, while the AlexaFluor647 signals were in blue. Seven distinct focal adhesions were boxed and labeled. The intensity of each of these focal adhesions over the widefield background was measured and presented in the table below the images. Widefield imaging was used for these measurements because it offers greater linear response than TIRF and does not have an out-of-focus cutoff of the image intensity. The images were well within the linear response of the instrument as the camera dynamics was less than half saturated.
(TIF)

Figure S5 CV1 transfected with EGFP-Talin1-mCherry were fixed, reacted with an anti-phospho-FAK antibody (anti-phosphotyrosine, T397, FAK), and stained with an AlexaFluor647 secondary antibody. Images were collected using a Deltavision Microscope. EGFP and mCherry signals were presented in green and red, respectively, while the AlexaFluor647 signals were in blue. Seven distinct focal adhesions were boxed and labeled. The intensity of each of these focal adhesions over the widefield background were measured and presented in the table below the images. Widefield imaging was used for these measurements because it offers greater linear response than TIRF and does not have an out-of-focus cutoff of the image intensity. The images are well within the linear response of the instrument, as the camera dynamics were less than half saturated and the instrument's response flat fielded. A long-lived single GFP-mCherry fluorochrome pair shows stretch and relaxation over multiple frames of observation.
(TIF)

Figure S6 Red-green separation upon stretch of a fluorochrome pair. The five frames show an increasingly high stretch of the molecule under observation. Pixels size is 81 nm and exposure time per frame 100 ms. The top row shows the raw TIRF data recorded, and the bottom row is the output of the cross-correlation of each channel with its point spread function plus a first order fit to suppress intensity fluctuations and background level (Bayesian match). Simulator data of the breakdown of the method. At low expression densities, the test scenario illustrated in Figure 3B provides an excellent estimate of how well the detection will perform at the envisioned expression densities. At higher and especially much higher expression densities, the method is, however, somewhat prone to aliasing. It might establisgh a very wrong estimate without detecting the failure.
(TIF)

Figure S7 Schematic dense arrangement of Talin. Molecules under observation get so close that the N-terminal of molecule A becomes a more likely “partner” of the C-terminal of molecule B than the N-terminal of molecule B, and hence it is wrongfully assigned. If there is a high density of label but not excessively high, the algorithm will pick up the “collision” by identifying the proximity of a second molecule, and it will discard that measurement. This leads to a mild artifact that we observed very regularly. The algorithm then limits itself to the areas of low expression and abandons the high expression zones. If, however, the density becomes much higher, the algorithm will no longer be able to segregate single molecules and in consequence sees mostly a homogenous background. In this case the findings of Figure 3B will no longer apply. Using the same test scenario with free termini leads to a very different distribution than the one of Figure 3B.
(TIF)

Figure S8 Simulation of measured distances between two tags in a dense, randomly distributed arrangement of fluorophores. Where Figure 3C constitutes the left tail of this distribution. The peak is reached at the mean free path between two identical molecules and the whole envelope forms a Poissonian distribution with lambda = “mean free path.” Beyond the mean free path, collision detection will no longer reliably work and the method fails catastrophically. In order to prevent such a scenario, we limit the background intensity to less than or equal to that of the observed single molecule. This limits the method safely to the left of the distribution and prevents aliasing. This limitation works locally without restriction, and hence some samples can at least be evaluated partially. The actin flow speed was measured in DIC to control the physiological behavior of the cells with the GFP-Talin-mCherry constructs.
(TIF)

Figure S9 Lamellipodium speed of GFP-talin1-mCherry transfected cell. DIC images in the top row and kymographs of fast ruffles in the bottom row; no drug, normal medium on the left; and added Y-27632 medium center, added blebbistatin on the right. CV1 cells were transfected with GFP-talin1-mCherry by lipofetamine2000 and were imaged after 48 h of transfection. Cells with positive transfection were confirmed by their fluorescence signal. Transfected cells were first imaged with normal growth media and then media containing Y-27632 (10 uM), followed by media containing blebbistatin (10 uM). Cell dish were washed with 1× PBS three times on the stage and incubated with normal growth medium for 30 min between each change of drug, also performed on the stage. DIC image series were collected on the Olympus IX81, 60× oil objective, 1.6 relays lens, photometrics K4, inside the Solent weather chamber and the Tokahit live cell incubator. Each image sequence recorded for 1 min at a frame rate of 1 per s. The inbound speed of the ruffles in the GFP-Talin-mCherry expressing cell in the left image was assessed to be 46 nm/s; 20 min after adding Y-Compound (center image), this decreased to 34 nm/s for the same edge; and finally, 40 min after adding blebbistatin, the movement nearly disappeared and decreased to 13 nm/s (image on the right). As on overview comparison, we measured the ruffle speed to be the following values for cells of the same time without the GFP-Talin-mCherry construct with normal medium: 53 nm/s, 60 nm/s, 51 nm/s, 24 nm/s, 30 nm/s, 29 nm/s, and 31 nm/s; and for other candidates with the constructs without drug treatment, the speeds obtained were 23 nm/s, 46 nm/s, 44 nm/s, 42 nm/s, 40 nm/s, and 43 nm/s.
(TIF)
