# Supplementary figures and images for: Mechanotransduction In Vivo by Repeated Talin Stretch-Relaxation Events Depends upon Vinculin
Source: PLoS Biol. 2011 Dec 20;9(12):e1001223. doi: 10.1371/journal.pbio.1001223 (PMC3243729; doi:10.1371/journal.pbio.1001223)

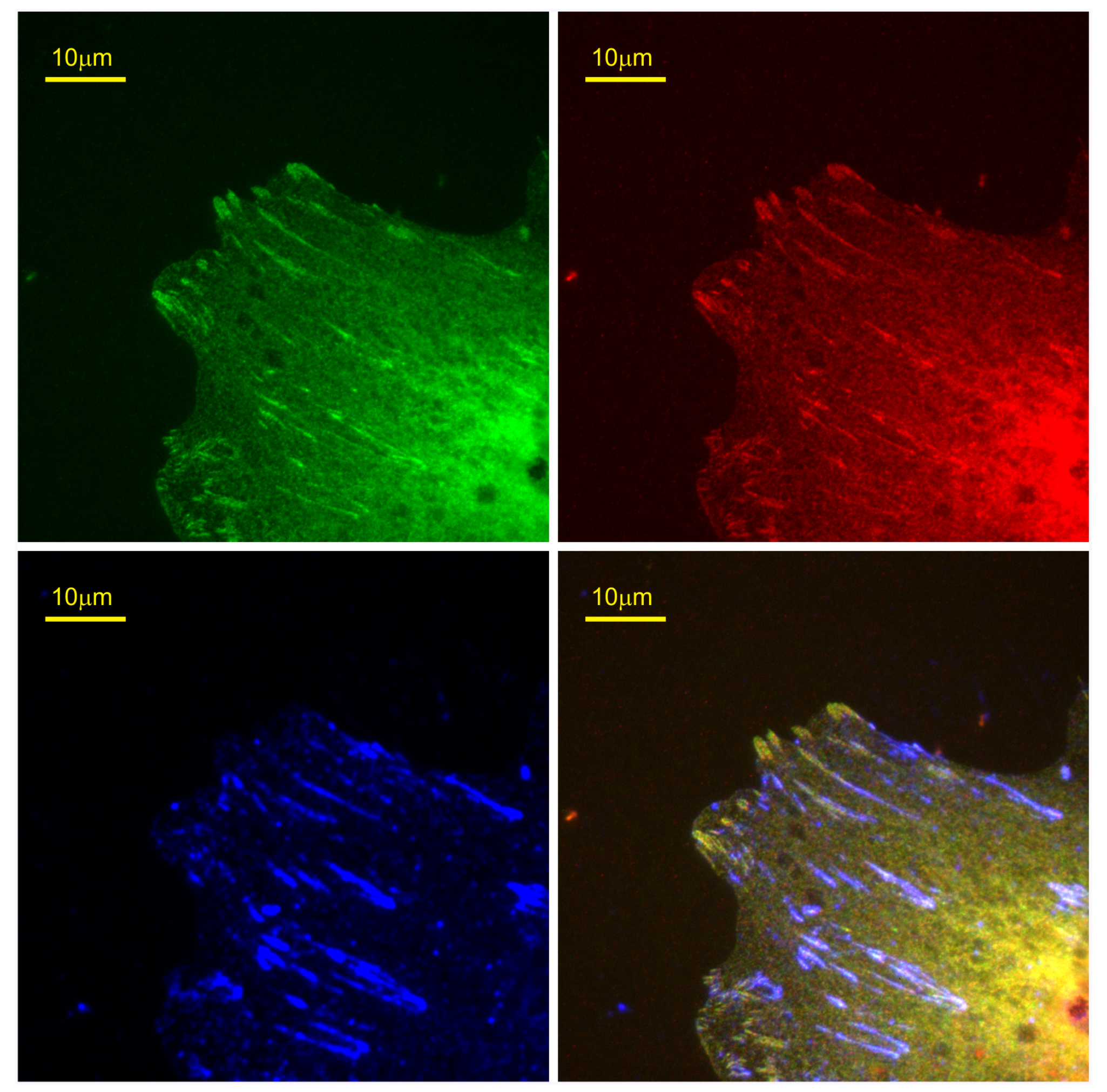

Supplement: Figure S1 — β1 integrin activation. Talin1 knockout cells were transfected with EGFP-Talin1-mCherry, fixed, probed with an anti-activated β1 integrin antibody, and stained with a secondary AlexaFluor647 antibody. Images were collected using a Deltavision Microscope. EGFP signals were presented in green, mCherry signals in red, while the AlexaFluor647 signals were in blue. (TIF) [file pbio.1001223.s001.tif]

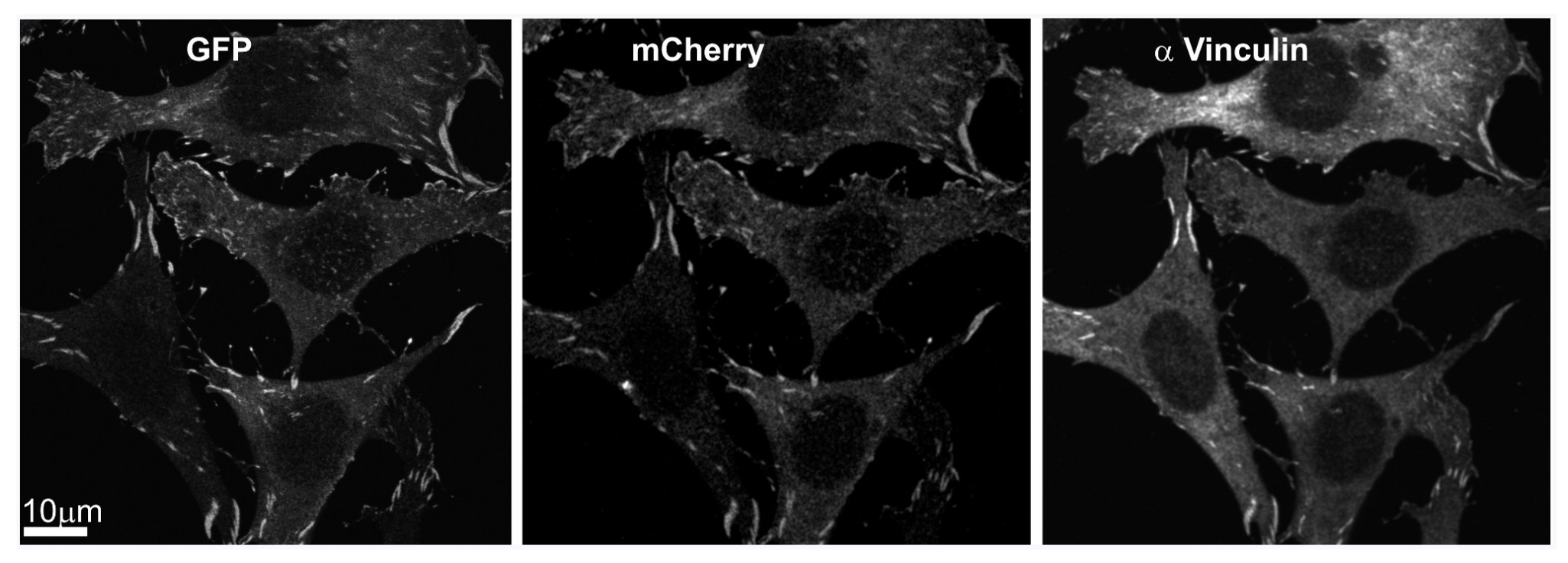

Supplement: Figure S2 — Expression of GFP-talin1-mCherry fully rescued the cell morphology and vinculin puncta formation in talin1−/− cells expressing talin2 siRNA (talin-depleted cells [13]). Cells were fixated after 60 min of spreading and stained for vinculin. This confirms that the GFP-talin1-mCherry construct behaves like natural talin when expressed in talin knockout cells, as they were capable of forming normal adhesions that also co-localized with vinculin. 1st plot, stretching of 2nd pair from the top; 2nd plot, stretching of a pair in the lower right; 3rd plot, trace at the leading edge; 4th plot, pair from the long adhesion in the center. (TIF) [file pbio.1001223.s002.tif]

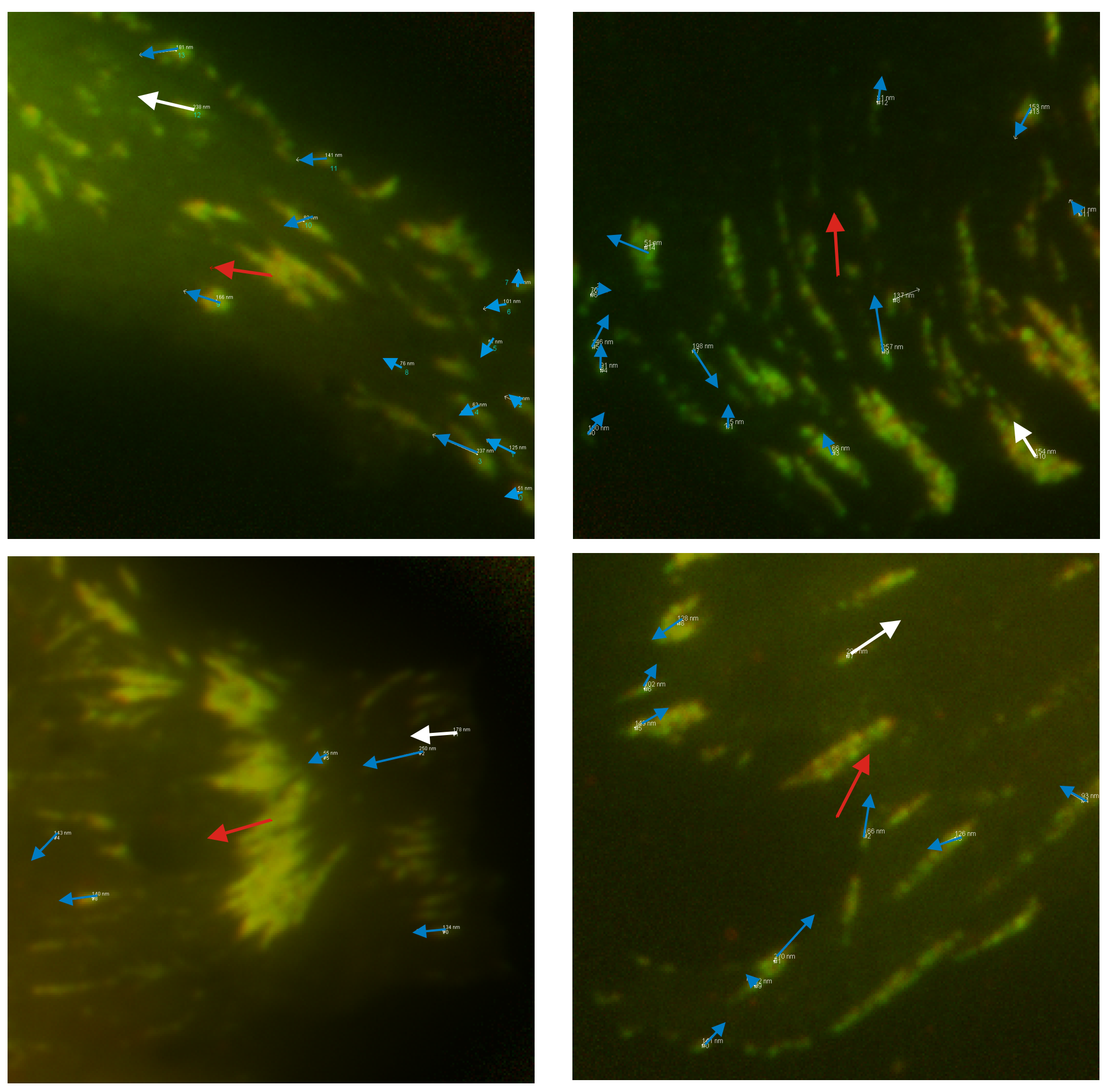

Supplement: Figure S3 — Talin single molecule observation over time. In Figure 3D we plot four distinct time courses of single fluorochrome pairs, each of which allowed observation over 60 s in 2 s time intervals (the arrows indicate the stretch of a pair at the snapshot time, white indicates the particular molecule we plotted, and red the average direction of all measured molecules). While GFP tags easily live in excess of hundred observations, mCherry proved to be more fragile, with the majority of the observations for an isolated pair being in the range of 15 to 20 frames. Imaging disruptions are, however, far dominant over fluorochrome blinking—if the PSF gets scattered, the frame drifts too fast, or another fluorescent signal comes too close during the observation, all this will interrupt the measurement. And even the successful observations in Figure 3D exhibit single frame drops where the localization proved not to be stable. Here we append the micrographs of the specimen from which these measurements were taken. (TIF) [file pbio.1001223.s003.tif]

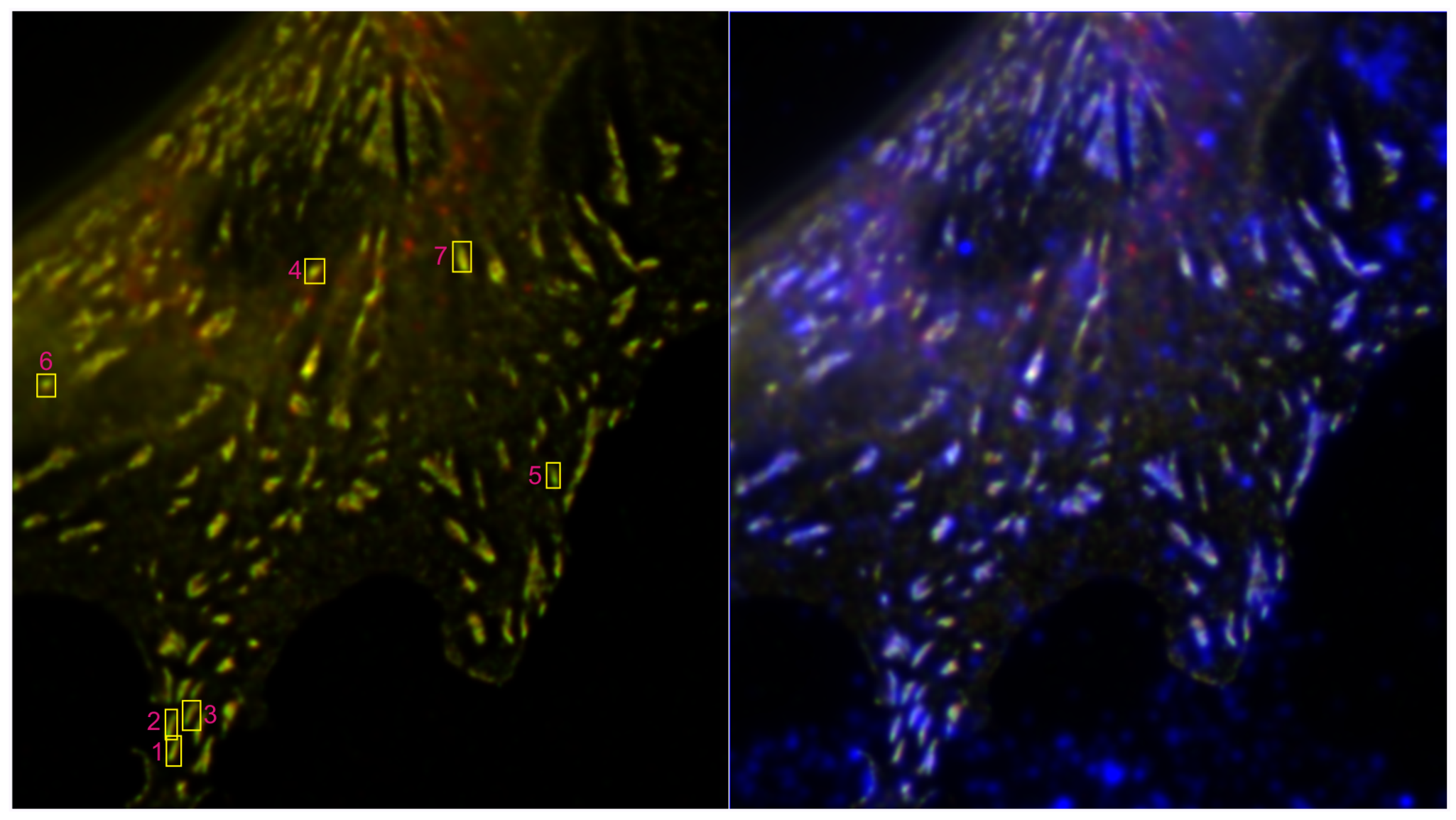

Supplement: Figure S4 — CV1 transfected with EGFP-Talin1-mCherry were fixed, reacted with an anti-vinculin antibody, and stained with an AlexaFluor647 secondary antibody. Images were collected using a Deltavision Microscope. Both EGFP and mCherry signals were represented in green and red, respectively, while the AlexaFluor647 signals were in blue. Seven distinct focal adhesions were boxed and labeled. The intensity of each of these focal adhesions over the widefield background was measured and presented in the table below the images. Widefield imaging was used for these measurements because it offers greater linear response than TIRF and does not have an out-of-focus cutoff of the image intensity. The images were well within the linear response of the instrument as the camera dynamics was less than half saturated. (TIF) [file pbio.1001223.s004.tif]

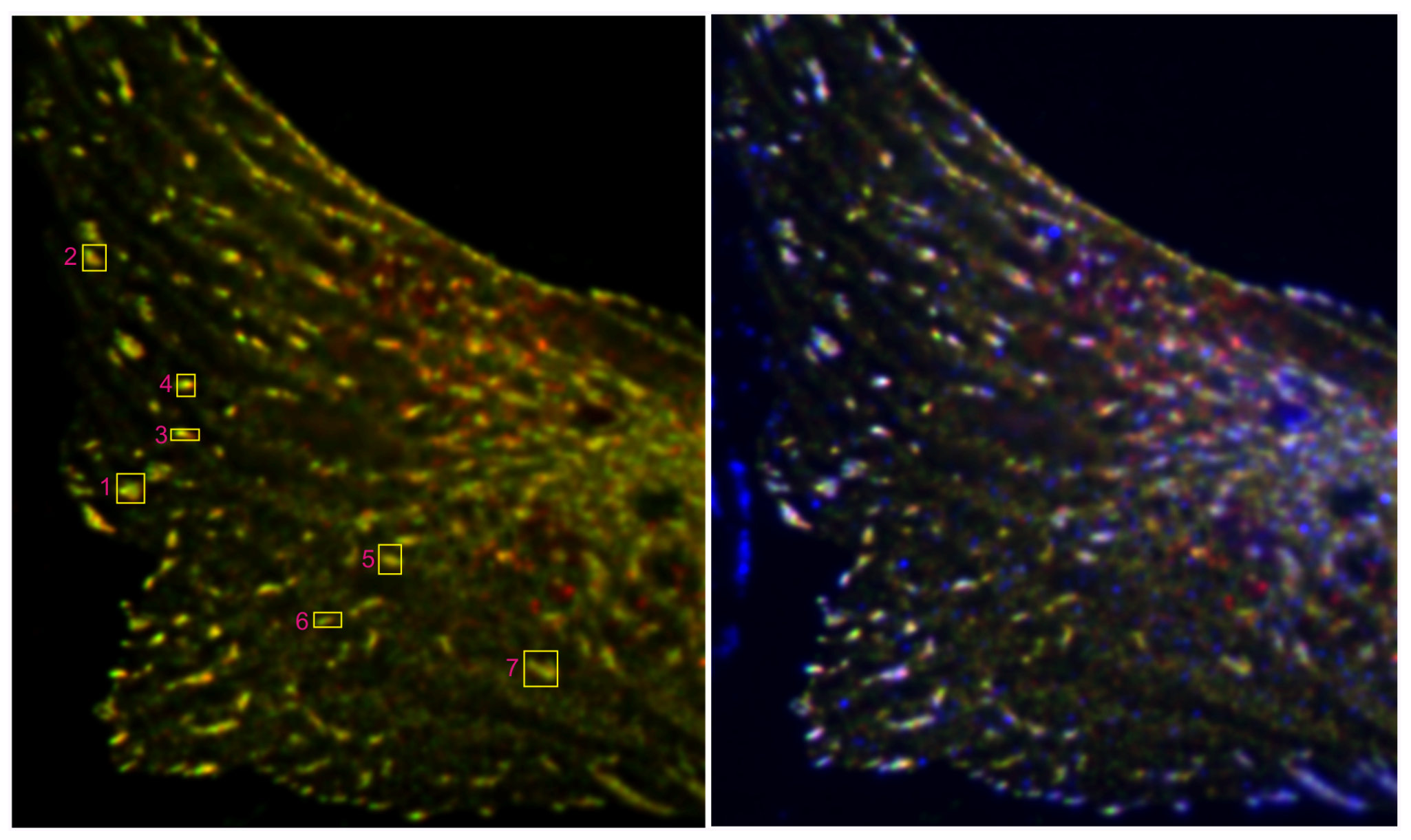

Supplement: Figure S5 — CV1 transfected with EGFP-Talin1-mCherry were fixed, reacted with an anti-phospho-FAK antibody (anti-phosphotyrosine, T397, FAK), and stained with an AlexaFluor647 secondary antibody. Images were collected using a Deltavision Microscope. EGFP and mCherry signals were presented in green and red, respectively, while the AlexaFluor647 signals were in blue. Seven distinct focal adhesions were boxed and labeled. The intensity of each of these focal adhesions over the widefield background were measured and presented in the table below the images. Widefield imaging was used for these measurements because it offers greater linear response than TIRF and does not have an out-of-focus cutoff of the image intensity. The images are well within the linear response of the instrument, as the camera dynamics were less than half saturated and the instrument's response flat fielded. A long-lived single GFP-mCherry fluorochrome pair shows stretch and relaxation over multiple frames of observation. (TIF) [file pbio.1001223.s005.tif]

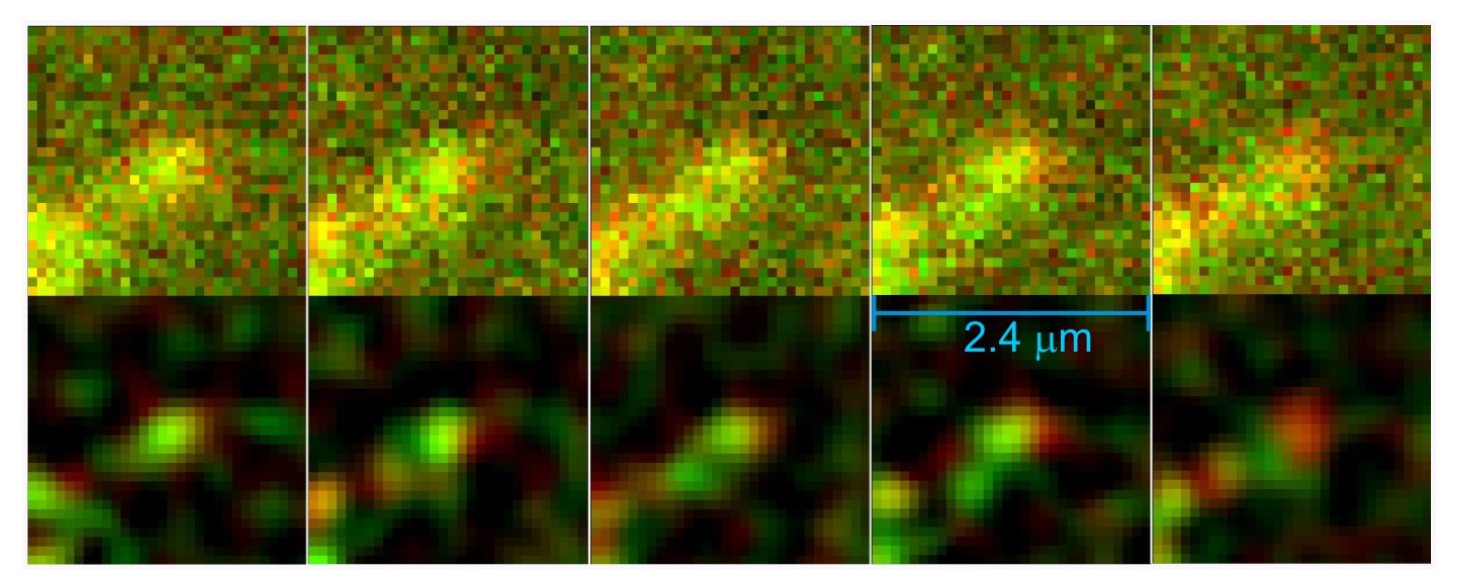

Supplement: Figure S6 — Red-green separation upon stretch of a fluorochrome pair. The five frames show an increasingly high stretch of the molecule under observation. Pixels size is 81 nm and exposure time per frame 100 ms. The top row shows the raw TIRF data recorded, and the bottom row is the output of the cross-correlation of each channel with its point spread function plus a first order fit to suppress intensity fluctuations and background level (Bayesian match). Simulator data of the breakdown of the method. At low expression densities, the test scenario illustrated in Figure 3B provides an excellent estimate of how well the detection will perform at the envisioned expression densities. At higher and especially much higher expression densities, the method is, however, somewhat prone to aliasing. It might establisgh a very wrong estimate without detecting the failure. (TIF) [file pbio.1001223.s006.tif]

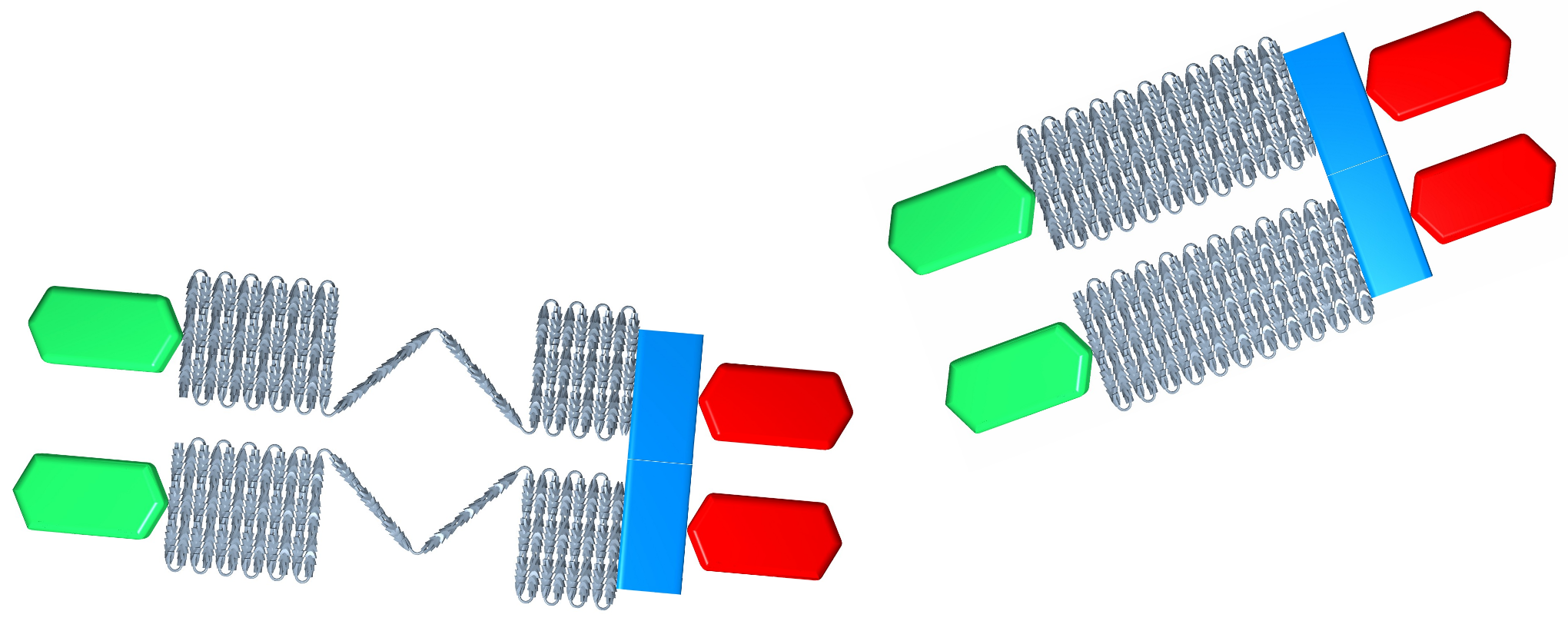

Supplement: Figure S7 — Schematic dense arrangement of Talin. Molecules under observation get so close that the N-terminal of molecule A becomes a more likely “partner” of the C-terminal of molecule B than the N-terminal of molecule B, and hence it is wrongfully assigned. If there is a high density of label but not excessively high, the algorithm will pick up the “collision” by identifying the proximity of a second molecule, and it will discard that measurement. This leads to a mild artifact that we observed very regularly. The algorithm then limits itself to the areas of low expression and abandons the high expression zones. If, however, the density becomes much higher, the algorithm will no longer be able to segregate single molecules and in consequence sees mostly a homogenous background. In this case the findings of Figure 3B will no longer apply. Using the same test scenario with free termini leads to a very different distribution than the one of Figure 3B. (TIF) [file pbio.1001223.s007.tif]

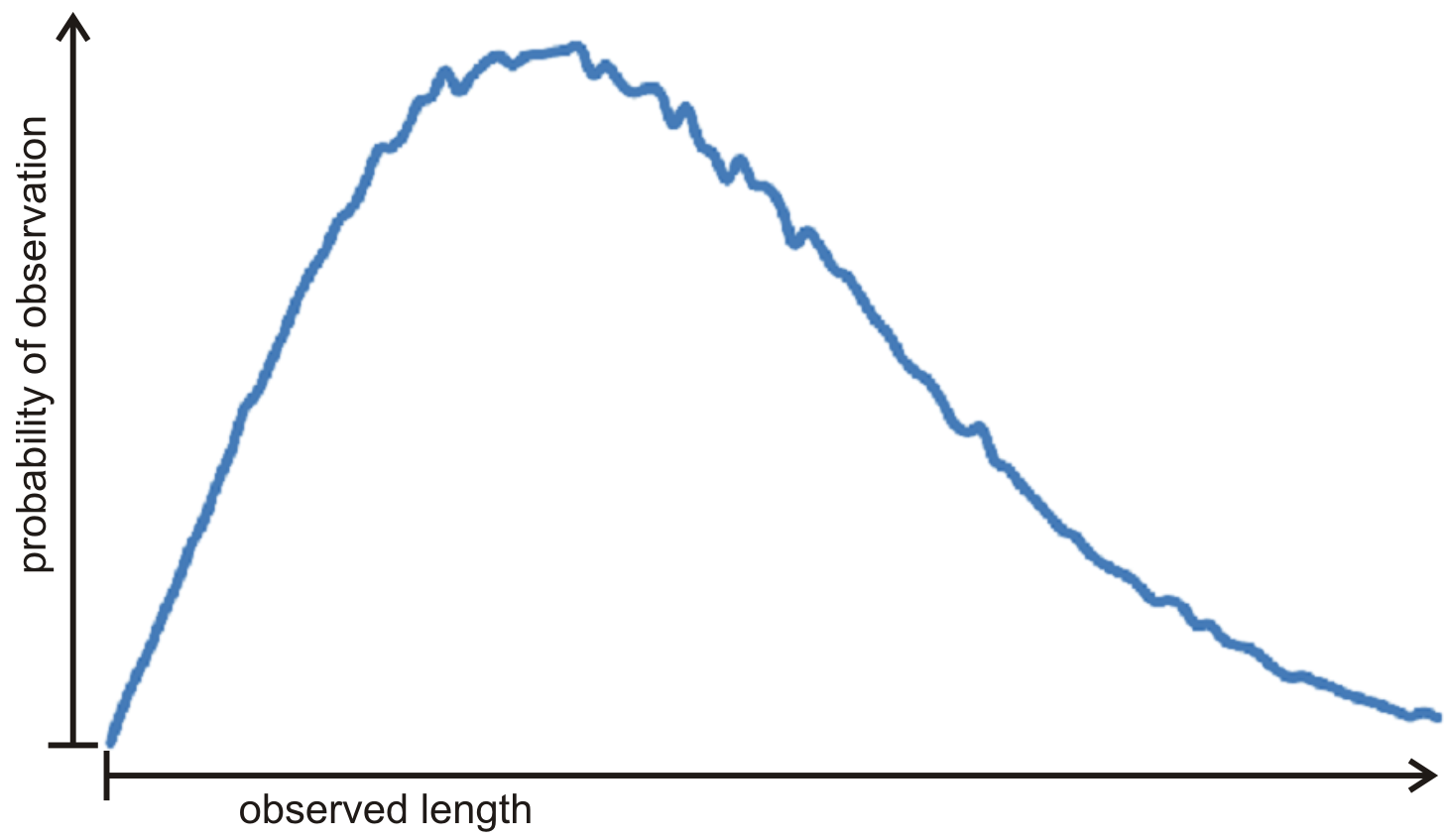

Supplement: Figure S8 — Simulation of measured distances between two tags in a dense, randomly distributed arrangement of fluorophores. Where Figure 3C constitutes the left tail of this distribution. The peak is reached at the mean free path between two identical molecules and the whole envelope forms a Poissonian distribution with lambda = “mean free path.” Beyond the mean free path, collision detection will no longer reliably work and the method fails catastrophically. In order to prevent such a scenario, we limit the background intensity to less than or equal to that of the observed single molecule. This limits the method safely to the left of the distribution and prevents aliasing. This limitation works locally without restriction, and hence some samples can at least be evaluated partially. The actin flow speed was measured in DIC to control the physiological behavior of the cells with the GFP-Talin-mCherry constructs. (TIF) [file pbio.1001223.s008.tif]

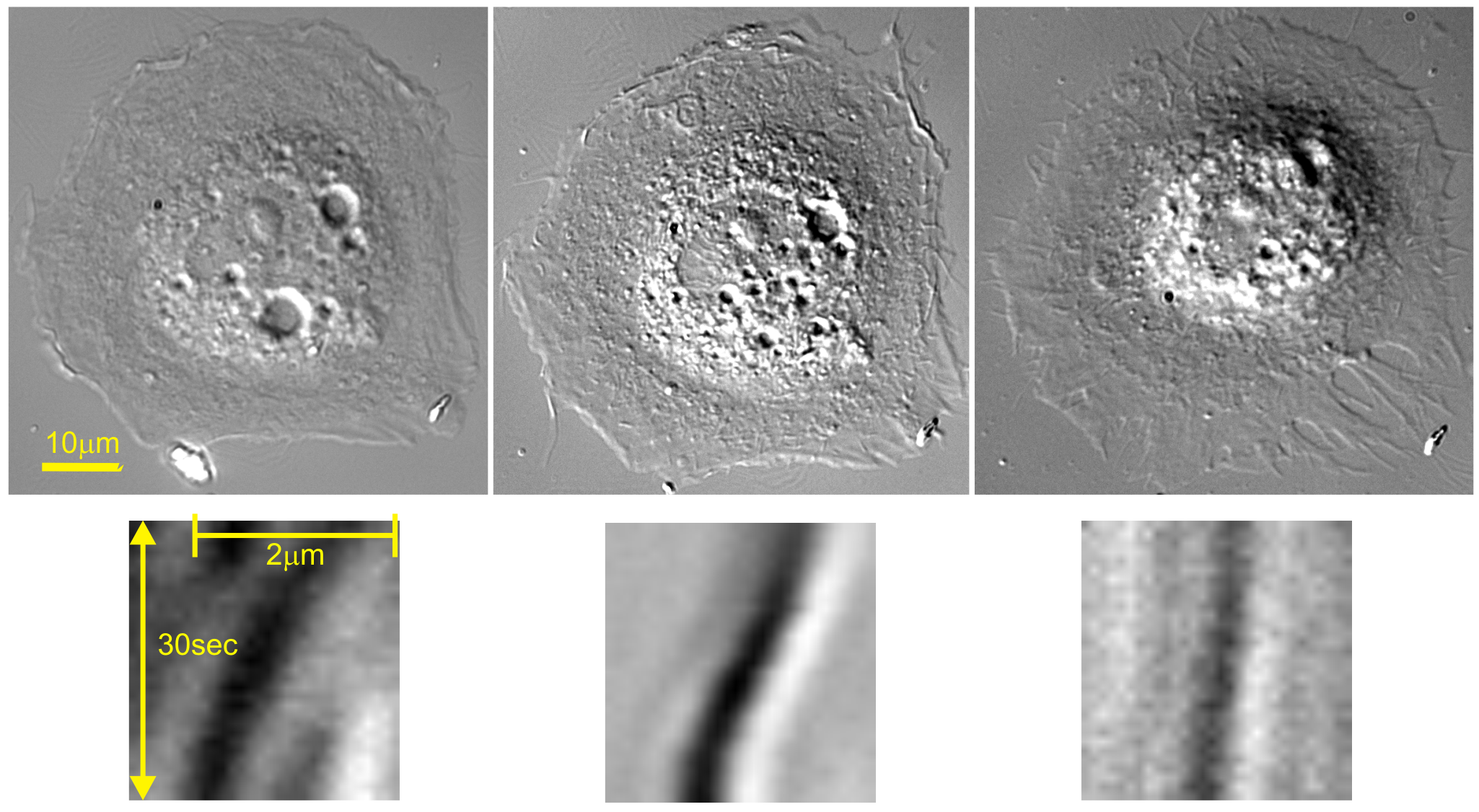

Supplement: Figure S9 — Lamellipodium speed of GFP-talin1-mCherry transfected cell. DIC images in the top row and kymographs of fast ruffles in the bottom row; no drug, normal medium on the left; and added Y-27632 medium center, added blebbistatin on the right. CV1 cells were transfected with GFP-talin1-mCherry by lipofetamine2000 and were imaged after 48 h of transfection. Cells with positive transfection were confirmed by their fluorescence signal. Transfected cells were first imaged with normal growth media and then media containing Y-27632 (10 uM), followed by media containing blebbistatin (10 uM). Cell dish were washed with 1× PBS three times on the stage and incubated with normal growth medium for 30 min between each change of drug, also performed on the stage. DIC image series were collected on the Olympus IX81, 60× oil objective, 1.6 relays lens, photometrics K4, inside the Solent weather chamber and the Tokahit live cell incubator. Each image sequence recorded for 1 min at a frame rate of 1 per s. The inbound speed of the ruffles in the GFP-Talin-mCherry expressing cell in the left image was assessed to be 46 nm/s; 20 min after adding Y-Compound (center image), this decreased to 34 nm/s for the same edge; and finally, 40 min after adding blebbistatin, the movement nearly disappeared and decreased to 13 nm/s (image on the right). As on overview comparison, we measured the ruffle speed to be the following values for cells of the same time without the GFP-Talin-mCherry construct with normal medium: 53 nm/s, 60 nm/s, 51 nm/s, 24 nm/s, 30 nm/s, 29 nm/s, and 31 nm/s; and for other candidates with the constructs without drug treatment, the speeds obtained were 23 nm/s, 46 nm/s, 44 nm/s, 42 nm/s, 40 nm/s, and 43 nm/s. (TIF) [file pbio.1001223.s009.tif]
